# Representation Biases: Variance Is Not Always a Good Proxy for Importance

**DOI:** 10.1523/ENEURO.0461-25.2026

**Published:** 2026-03-05

**Authors:** Andrew Kyle Lampinen, Stephanie C. Y. Chan, Yuxuan Li, Katherine Hermann

**Affiliations:** Google DeepMind, Mountain View, California 94043

**Keywords:** artificial neural networks, computational neuroscience, deep learning, representation analysis, statistical methods

## Abstract

A central approach in neuroscience is to analyze neural representations as a means to understand a system's function, through the use of methods like principal component analysis, regression, and representational similarity analysis. These analyses often rest on a tacit “linking assumption”: that the features explaining the most variance in neural activity are the most important for the system's computation. Here, we challenge this assumption. We review recent work in machine learning demonstrating “representation biases”—the fact that learned representations can be biased toward certain features over others. For example, learned representations heavily overrepresent simple (linear) features while representing complex (nonlinear) features much more weakly, even when both are equally critical for the system's computations. We review the origins of these biases in learning dynamics and patterns of computation. We then discuss their consequences for neuroscience. We show that if a subset of features dominates the representations, standard analytic techniques can yield highly biased inferences—for example, resulting in the mistaken conclusion that a system is simpler than it really is or that two systems are more similar than they really are. We discuss some connections between these findings and recent empirical developments in neuroscience. Finally, we present homomorphic encryption as a conceptual case study of the potential for a total dissociation between representational geometry and computation. We conclude that achieving a complete understanding of neural systems requires moving beyond high-variance signals, as critical computational mechanisms may be hidden in low-variance components.

## Significance Statement

Computational neuroscientists often attempt to understand the brain by analyzing patterns of neural activity. These analyses frequently rely on the assumption that the strongest signals—those that explain the most variance—reflect the most important computations. This work challenges that core assumption. Using artificial neural networks where the computational “ground truth” is known, we demonstrate that learned representations are systematically biased toward some features over others. For example, simple linear features generate strong, distinct signals, while complex, nonlinear computations are frequently hidden in low-variance activity. Consequently, standard analytic tools may be biased toward identifying certain aspects of a system’s computation. Thus, these findings highlight challenges for achieving full understanding of a system's computations using common methods.

## Introduction

A central approach of neuroscience is analyzing patterns of neural representation to learn about a system (Kriegeskorte and Diedrichsen, 2019). In particular, computational neuroscience has increasingly relied on relating patterns of neural activity to the internal representational structures of computational models (Churchland and Sejnowski, 1990; Kriegeskorte et al., 2008; Feather et al., 2025; Sucholutsky et al., 2025). However, there are philosophical questions about how to justify interpreting internal activity as representations (Shea, 2018; Cao, 2022b; Fallon et al., 2023) and conceptual and practical challenges to understanding a system through analyzing its internal activity or representations (Poldrack, 2006; Marom et al., 2009; Jonas and Kording, 2017; Ritchie et al., 2019; Sexton and Love, 2022; Dujmovic et al., 2024).

**Figure 1.**
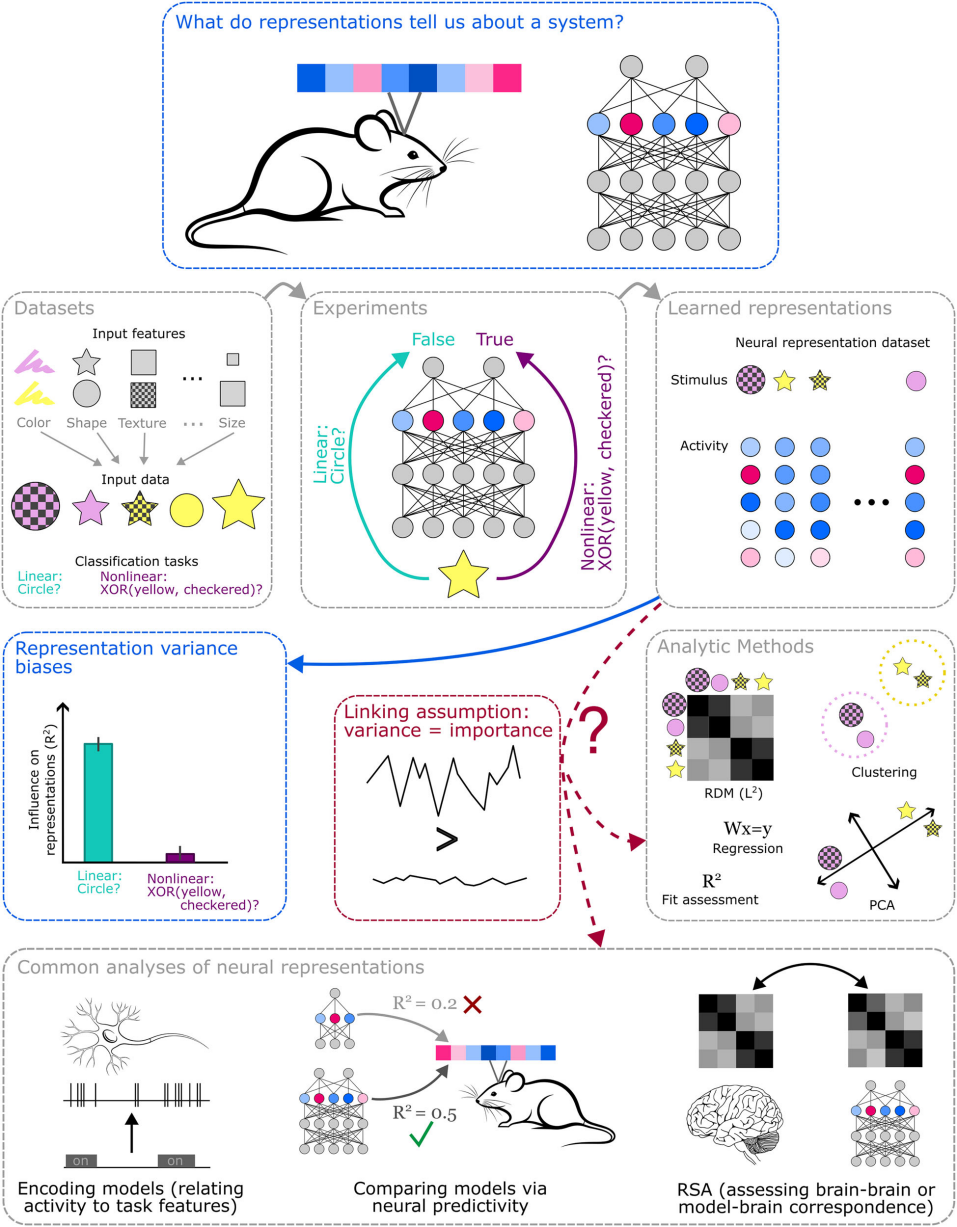
What do representations tell us about a system? Here, we discuss some experiments that illustrate challenges in making inferences about a system from its representations. The experiments involve training models on controlled datasets mapping from many input features to outputs involving simpler or more complex functions of the input features. The representations that models learn from these datasets show substantial biases toward certain outputs over others (e.g., toward simple linear functions of the input over complex nonlinear ones), even when the model has reliably learned both. These biases raise questions about a linking assumption that underlies many analytic methods and common analyses in neuroscience: that variance explained in the neural representations (or activity) is a good proxy for the importance of a signal.

Here, we use case studies from recent work in machine learning to illustrate some practical challenges to understanding a system's function by studying its internal representations (see Fig. 1 for an overview) and discuss the implications for neuroscience. In particular, we focus on the results of Lampinen et al. (2024). In this work, the authors study the relationship between patterns of representation and computation in machine learning models, using controlled experiments. The authors identify substantial biases in the learned representations: some features are much more strongly represented than others, even if they play similar computational roles in the system's behavior. These representation biases mean that common analytic methods—such as principal component analysis (PCA), representational similarity analysis (RSA; Kriegeskorte et al., 2008), and linear regression—may be biased toward capturing some computational features over others. Indeed, we review results from Hermann and Lampinen (2020) that illustrate some of these potentially surprising findings. Thus, the many types of neuroscience experiments that use methods like these to study a system's representations or activity may provide a biased picture of its computations.

We first briefly review the prior literature on representations and computations in cognitive science and neuroscience. We then turn to our central examples illustrating representation biases and how they impact common representational analyses. We then briefly present a case study of homomorphic encryption, which illustrates how strong the dissociation between representations and the system's function can be. We close by discussing the implications of our results and the questions they raise.

### Background

In a foundational paper, Marr (1982) famously posited three levels of analysis for understanding an information processing system—computational, algorithmic, and implementational. In the years since, there has been substantial discussion about the (non)independence of the levels, their interactions and joint constraints, and what they imply about representation (Churchland and Sejnowski, 1990; McClamrock, 1991; O’Brien and Opie, 2009; Stevens, 2012; Piccinini and Shagrir, 2014; Hardcastle and Hardcastle, 2015). In the philosophical literature, similarly, the question of the relationship between representation and computation has been somewhat fraught (Eliasmith and Anderson, 2003; Piccinini, 2008; Van Gelder, 2014; Shea, 2018).

There have also been arguments from a variety of perspectives that intelligence is best understood (or engineered) by focusing on the interaction between a system and its environment (Brooks, 1991; Clark, 1998; Cisek, 1999; Varela et al., 2017). These approaches therefore tend to think of “representations” as more dynamic and entangled with the environment state rather than as static ways of encoding information.

In addition to the conceptual debates about representation, there has been substantial debate about the empirical relationship between the representations of a system and its computational mechanisms or behavior. Several works have highlighted particular case studies where applying neuroscience analyses to well-understood computational systems does not yield the expected results (Marom et al., 2009; Jonas and Kording, 2017). In machine learning, aside from the main paper we discuss, a number of other works have demonstrated that there can be substantial dissociations between the internal representational structure of a system and its computations or behavior (Hermann and Lampinen, 2020; Braun et al., 2025)—especially under distribution shift (Friedman et al., 2023; Dujmovic et al., 2024).

There has also been a longer-standing discussion about the more concrete issue of the relationship between neural activity and cognitive mechanisms. For example, many researchers have noted that making inferences about cognitive processes or information content from downstream effects like fMRI activity (Poldrack, 2006) or decoding (Ritchie et al., 2019) can be logically invalid. Others have noted the possibility that representational correlations do not necessarily imply computational correspondence and therefore emphasized more stringent measures (Sexton and Love, 2022). As in the conceptual work above, some neuroscientists have taken more provocative stances that “representation” is generally the wrong way to understand neural activity (Freeman and Skarda, 1990).

However, there is clearly still information to be gained from representational analyses, and thus many researchers take a pragmatic perspective on representation in neuroscience: interpreting activity measures as a representation insofar as it satisfies conditions of reliable decodability together with causal efficacy (Cao, 2022b; cf. Poldrack, 2021; Baker et al., 2022). That is, a pattern (or distribution) of activity can be considered a representation insofar as we can consistently decode the expected information content from it, and intervening on it changes the system's behavior in the expected way—e.g., if we can identify a consistent pattern of neural activity corresponding to a stimulus, and reactivating that representation produces the stimulus response even when the stimulus is absent. We adopt a similar perspective here, and the “representations” studied here satisfy both criteria.

While the issues we focus on here are related to some of these overlapping discussions, we focus more on the question of how we identify candidate feature representations in the first place—and whether the types of feature representations that we identify might be systematically biased toward those that serve certain kinds of computations.

## Materials and Methods

Here, we briefly summarize the relevant methods used by the machine learning works that we review (Hermann and Lampinen, 2020; Lampinen et al., 2024). The results in question involved analyzing the learned representations of artificial neural networks.

### Models

The results we discuss here focus on deep multilayer perceptron (MLP) models of four to five layers, with large representation dimensions (e.g., 256 hidden units per layer) relative to their output size (one to two outputs). The models include rectifying nonlinearities at each hidden layer, which are commonly used in modern machine learning, and have been argued to be relatively biologically plausible as models of firing rates (Glorot et al., 2011). The prior works also document similar results in a wider range of settings, including deeper models, as well as other networks of contemporary interest, such as convolutional networks and transformers on image and language inputs. Here, we focus on the MLP experiments for brevity.

### Tasks

The tasks discussed here involve moderately sized binary input vectors (32 inputs), from which the model is to independently perform multiple classification tasks simultaneously through distinct output units (i.e., a multitask setting). Each classification task depends on a set of input units that are disjoint from those relevant for other tasks; thus, the input and output units for each task are completely disjoint, so given that the architectures have large hidden layers, there is no a priori reason that the tasks should interact. There are also a larger number of “noise” inputs that do not contribute to either task.

In particular, the results we discuss here focus on a setting in which the models are trained to compute an “easy” (linear) and “hard” (nonlinear) classification of the binary input vectors. The easy, linear feature corresponds to reading out the value of a single input unit. The hard, nonlinear feature is 4-parity: a more difficult version of XOR that can be interpreted as deciding whether the sum of four binary inputs is an odd number.

### Training

The model is trained by a standard sigmoid cross-entropy classification loss via stochastic gradient descent (SGD). Again, the prior works documented similar results with other optimizers. The models are trained on a sufficiently large dataset that they generalize perfectly to a held-out test set for either feature; thus, the models are computing these two functions in a way that generalizes reliably.

### Internal representations

The representational analyses focus on the learned representations at the penultimate layer of the model, immediately before the final binary classification. Given that the models are solving the tasks in a way that generalizes (as noted above), all output variables must be linearly decodable from this layer. However, this does not mean that they are equivalently represented. (Note that in some experiments the prior papers also considered how features transform across different layers of the model, e.g., from encoding to decoding phases in a language model.)

### Representation variance

The main analyses of representations used in the work are encoding models—that is, linear regressions from the values of individual task features onto the model's internal representations. The primary quantification of interest is the variance explained (R^2^) in the internal representations by each of the task features, computed on a holdout set after fitting the regressions on a training set.

Some subsequent analyses use augmented encoding analyses that account for different ways that the nonlinear tasks can be represented. To do so, these analyses regress from a larger set of features (e.g., from all the 2^4^ = 16 features that could be relevant to computing the 4-parity task)—in this case, these different features are effectively treated as a categorical (one-hot) predictor. From this regression, variance explained can likewise be calculated on the holdout set as before; assuming appropriate regularization, the variance explained by this measure should always be strictly greater than that explained by the single-task-variable encoding measure.

Variance explained in the representations is an important variable because it has downstream implications for a number of common analyses such as PCA and RSA; these analyses are likewise performed on the same sets of representations from the same penultimate layer.

### RSA

The RSA results discussed here used Euclidean *ℓ*^2^ distance as the representational similarity metric and Pearson’s correlation to quantify similarities of the dissimilarity matrices; however, Hermann and Lampinen (2020) and Lampinen et al. (2024) both report similar results with other representational distance measures like cosine distance and with other methods for quantifying similarity.

### Code availability

Lampinen et al. (2024) published code for reproducing the easy versus hard feature bias effects at https://gist.github.com/lampinen-dm/b6541019ef4cf2988669ab44aa82460b.

## Results: Representation Biases

**Figure 2.**
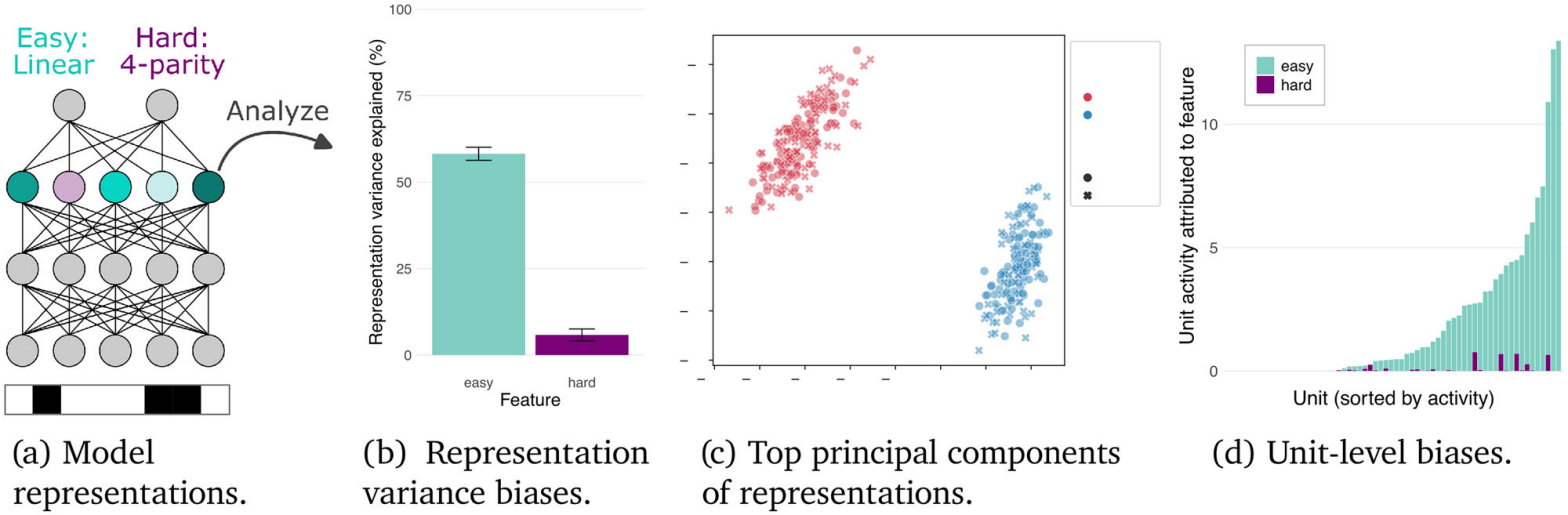
Model representations can be substantially biased toward certain features, in the sense that those features carry much more variance in the representations than others that have similar computational roles. These analyses focus on the representations at the penultimate layer of an MLP model (***a***) trained to independently classify two features of a binary-valued input vector: one easy linear feature and one hard highly nonlinear feature. Although the final-layer task of the model is the same for both features—binary classification from the penultimate representation—the model's penultimate layer representations are heavily biased toward the easier feature (***b***), with over 50% of the variance in these penultimate representations explained by the easy feature and only ∼5% by the hard feature. Thus, in the top principal components (***c***) the representations are clearly clustered by the easy feature (colors), but the hard feature value (shapes) does not have a clear influence. At the individual unit level (***d***), the biases are equally clear—most of the units respond to the easy feature, and even those units that encode information about the hard feature tend to have more of their activity explained by the easy feature.

Here, we introduce the results of Lampinen et al. (2024). One kind of bias explored by the authors is depicted in Figure 2. A simple MLP model is trained to perform independent binary classification on an “easy” (linear) and “hard” (nonlinear) feature of binary input vectors.

Although the model is performing the same type of output classification on both features—i.e., a final linear classification—the variance explained in the representations by the easy feature is an order of magnitude higher than that explained by the harder feature.

Furthermore, this bias is also evident at the single-unit level. More units encode information about the easy feature, and even those that encode information about the harder feature still tend to have more of their variance explained by the easy feature—especially for the most active units.

Because of this variance bias, when analyzing this model's representations, many analyses tend to be biased toward the easy feature. For example, PCA identifies precisely the dimensions in the representations that carry the most variance. Thus, the first several PCs show clear clusters according to the easy feature and little organization according to the more difficult feature.

**Figure 3.**
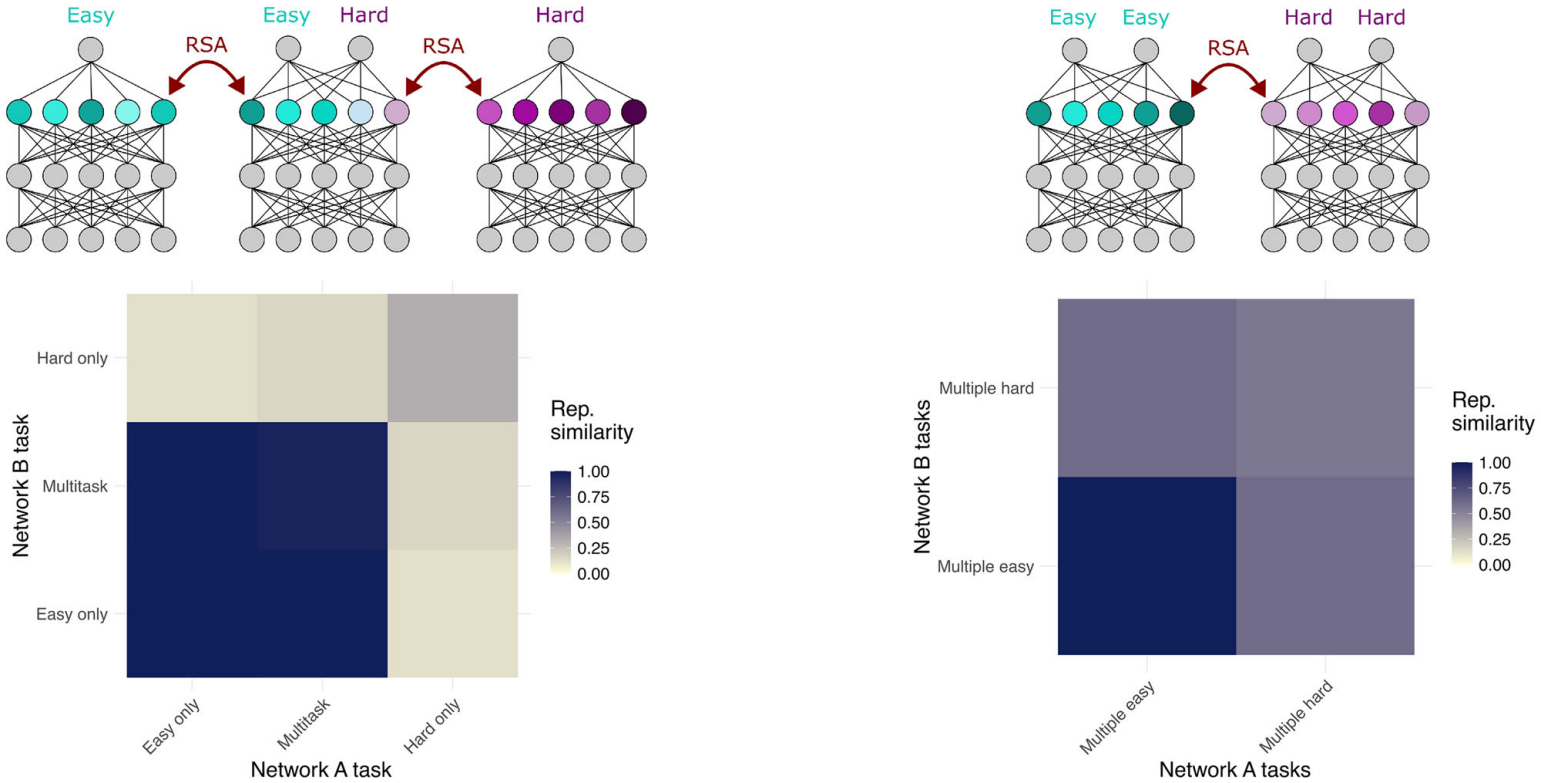
Representation biases have downstream impacts on analyses that compare representations between systems. Here, we show that RSA within and between different sets of models can give surprising results due to representation biases. In each plot, ideally the blocks on the diagonal would be darkest (i.e., show the highest similarity), and the blocks off the diagonal would show graded similarity corresponding to the functional overlap. However, that is not the case. Left, When comparing a model trained to output both easy and hard features to ones that are trained on only one feature, the multitask model appears very similar to the easy-task-only model (Hermann and Lampinen, 2020). In fact, the models trained only on the hard task do not even appear particularly similar to other models trained on the same exact task. Right, When models are trained on multiple easy or multiple hard tasks, the models trained on only hard tasks appear less similar to other models trained on exactly the same tasks than they do to models trained on strictly easier tasks that use the same input units (5 models were trained for each combination of features).

Beyond PCA, many analyses treat variance as a proxy for the importance of a signal. Thus, these biases also affect other analyses like regression (onto the unit level or population representations, e.g., from task features, a computational model, or another neural system) or RSA. Figure 3 demonstrates how the biases can give surprising results in RSA. For example, when a model that is trained to compute both easy and hard features is compared with separate models that compute only the easy feature or only the hard feature, the representations of the multitask model appear very similar to those of the easy-task-only model and very different from those of the hard-task-only model (cf. Hermann and Lampinen, 2020). In some cases, models that are trained to compute only hard features can appear less similar to other models trained on the same tasks than they do to models that are computing easy features from overlapping inputs. Thus, the models that have the most similar representations are not necessarily the ones that are computing the most similar functions. These results are supported by several other recent machine learning studies that similarly highlight the possibility for dissociations between representation and computation (Friedman et al., 2023; Braun et al., 2025).

### Why are representations biased?

**Figure 4.**
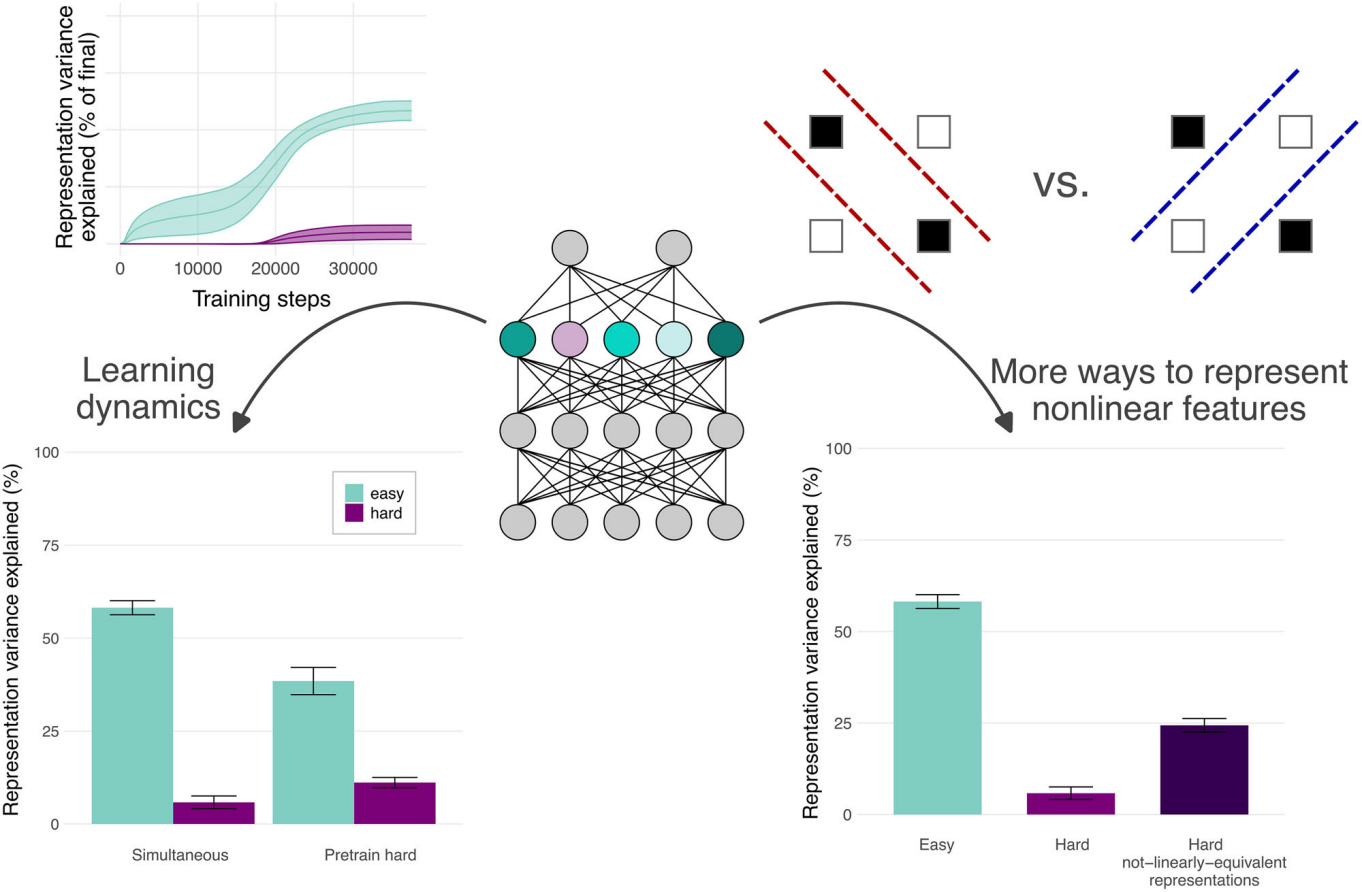
Why are representations biased toward easier features? The biases are driven by multiple factors, including learning dynamics and the different ways that nonlinear features can be represented. Left, By manipulating training order (training the hard task first rather than both simultaneously), the magnitude of the biases can be reduced. Right, Likewise, by accounting for the fact that there can be more ways to represent a nonlinear feature that are not linearly equivalent—for example, different ways of drawing intermediate classification boundaries to compute an XOR function—we can identify other components of the representations that may be contributing to the model's computation of the hard feature. Together, the learning dynamics and multiple ways of representing features explain most of the representation bias toward the easy (linear) feature over the hard (nonlinear) one.

Lampinen et al. (2024) also investigate the origins of the above biases; their conclusions are illustrated in Figure 4. In short, multiple factors contribute to the biases. The most straightforward factor is that features that are learned first tend to be favored in the representations. Simpler features tend to be learned before more complex ones (cf. Shah et al., 2020; Huh et al., 2021), and this bias has been suggested to be a contributor to the generalization of deep learning (Huh et al., 2021). This pattern can also be seen as an instance of the “availability” biases highlighted by Hermann et al. (2024). Indeed, Lampinen et al. (2024) showed that other features that change which features are learned first (such as the prevalence of a feature in the dataset) also drive similar representation biases. When learning order is reversed by pretraining the hard feature, the representational gap between the easy and hard features narrows (Fig. 4, bottom left). However, learning order does not fully explain the difference between the two features.

The other factor is that linear and nonlinear features yield different patterns of representation. All the natural (e.g., the ways that a feature could be represented in the minimum-capacity two-layer network that computes it) ways of representing a linear feature are equivalent up to linear transformation. In contrast, there can be more ways to represent a nonlinear feature that are not equivalent to one another. For example, to compute the XOR function, there are several ways of drawing the classification boundaries (Fig. 4, top right) that yield different representational similarity structures—these different modes of representation discard different pieces of information, so it is not possible to (linearly or nonlinearly) transform from one to the other with complete accuracy. To detect the effect of these different patterns of representation, the authors regress from an augmented space from which all representation patterns are linearly decodable. Indeed, accounting for multiple patterns of representation explains part of the gap between the linear and nonlinear features. In high-dimensional representation spaces, these multiple not-linearly equivalent solutions tend to dilute the variance due to a computation across more representational dimensions that vary in dissimilar ways, resulting in patterns of activity that are less similar between models trained on the same task and less variance attributable directly to the nonlinear feature in question.

It is natural to ask whether the differences in representation patterns for the different types of features should really be interpreted as a “bias”—after all, they do correspond to meaningful differences in computations (and how they map onto other levels of analysis) between the two features. We will return to this issue in the discussion. However, regardless of how they are interpreted, these differences still lead to biases in our inferences from representations; e.g., when using analyses like RSA or PCA, they lead us to focus on representations used for computing only some of the features a system is using.

### The generality of feature biases

While for brevity we focus on biases due to feature complexity here, Lampinen et al. (2024) also find similar biases driven by other factors like prevalence in the data or position in a time sequence. Furthermore, while the examples here are taken from MLPs, there are similar types of biases in transformers and convolutional networks. Furthermore, the biases generally persist under various optimizers, regularization like dropout or weight decay, etc. Concurrent works have also observed related biases in larger vision models trained on more naturalistic data (Fel et al., 2024)—and the representations of large language models tend to be dominated by linear features (Park et al., 2024), despite the fact that the models weakly represent many nonlinear features (Marks, 2024). Thus, these biases seem to be a fairly general phenomenon of feature learning.

## How Might Feature Representation Biases Impact Neuroscience?

Given that neuroscience often relies on analysis of representations, the kinds of biases discussed above could impact inferences in neuroscience as well.

For example, a growing area of research relies on comparing representations between AI models and humans or animals (Sucholutsky et al., 2025), and there have been explicit calls for studying representational as well as behavioral alignment of models to the brain (Feather et al., 2025)—as well as collaborative benchmarks for this purpose (Schrimpf et al., 2020). However, if the AI models are biased toward representing certain features more strongly, this could lead to biased inferences from these analyses and benchmarks. For example, such biases might help to explain why there is surprisingly little difference between different AI model architectures in how closely they align with brain representations (Conwell et al., 2023)—if the representations of the various models tend to be dominated by the simplest (or most prevalent) features which any objective will learn.

However, even for other types of analyses, the existence of representation biases raises questions. Do natural systems similarly produce representations that are biased by these (or other) factors? If so, then the kinds of computations and similarities that we tend to discover in these systems may likewise be biased. For example, we might overestimate how much high representational alignment between two humans (or animals) tells us about the alignment of their computations—the systems might have high representational alignment even if they only align on some features.

Conversely, it is common practice to compute a noise ceiling for comparisons by using between-individual predictions, e.g., using between-subject neural predictivity as an upper bound on how well a model could be expected to predict neural data. However, the fact that some features can be computed in ways that yield nonequivalent patterns of representation means that interpreting such measures as a “noise” ceiling could be misleading. Some of the features that each representation fails to predict in the other may be important parts of the computational solution that the two systems simply represent in incompatible ways. For example, in real data for complex tasks like language comprehension, a few (important and interesting) dimensions are shared across individuals (Tuckute et al., 2025); however, the majority of the within-subjects explainable variance is not captured by these shared dimensions. Thus, a model that can predict the representations “as well” as the between-subjects noise ceiling might nevertheless miss some of the important computational features. The right panel of Figure 3 shows a concrete example of how representation biases can contribute to this effect, where the multiple-easy model matches the representations of the multiple-hard models better than the multiple-hard models match each other—but the representations of the multiple-easy model do not solve the hard tasks, whereas the less-aligned features of the multiple-hard models all consistently solve the same tasks.

Biased representations could also introduce challenges for analyses at lower levels. For example, in the experiments above, the units whose activity was most driven by the hard feature still tended to carry much stronger signals about the easy feature (Fig. 2, right). Thus, these units might still get clustered with the units that only encode easy signals or get removed from analyses due to their mixed selectivity (cf. Rigotti et al., 2013). If natural systems have similar unit-level biases, that would therefore similarly raise questions about whether the types of features we tend to identify a unit as encoding are necessarily the only important features about which that unit carries information.

Indeed, several recent neuroscience papers have highlighted challenges to the idea that low-dimensional, high-variance components of the representations are sufficient for characterizing the system. Gauthaman et al. (2025) show that, when characterized appropriately, human visual representations show thousands of representational components (within and across individuals) whose explained variance follows a power law; they therefore argue that focusing on low-dimensional representations may be inadequate for explaining all aspects of visual processing. Pereira-Obilinovic et al. (2025) show causally that in a predictive model of neural behavior, components outside the low-dimensional coding dimensions play a causal role in driving decisions—indeed, more reliably than those in the lower-dimensional coding subspace. Our work complements these empirical findings by showing, in a controlled setting, how lower-variance components may end up carrying qualitatively different types of information and thus why neglecting them may be especially problematic.

Thus, representational biases could pose challenges for a range of common analyses. Of course, even with biases, there is still much that can be inferred from representational analyses; we will return to this point in the discussion. First, however, we will consider a final, more extreme case study.

## Homomorphic Encryption: Strongly Dissociating Computation from Superficial Patterns of Representation

In the experiments described above, the role that the representations played in the computations of the system was relatively straightforward, even where the representations were biased toward one feature. However, this does not have to be the case. We illustrate this with a final conceptual case study of the possibility for strong dissociation between computation and patterns of representation: homomorphic encryption (Gentry, 2009; Van Dijk et al., 2010). While the field of cryptography is largely focused on creating representations that preserve information yet are not easily decodable, in homomorphic encryption schemes it is additionally possible to perform arbitrary computations (any algebraic circuit) over this information while it is encrypted. That is, at each step of such a computation, a new encrypted representation is produced that, when decrypted, yields the representation at that step of the original computation.

This example shows that it is not necessary for a computational system to have any straightforward (e.g., linearly decodable) format of representation of the features that it uses in its computations. Systematic computations can be performed even over representations that are deliberately crafted to thwart attempts to understand (decrypt) their content.

As a special case, this example also illustrates that systematic compositional computations are possible without requiring representations that are straightforwardly (e.g., concatenatively) compositional in their original format. Encrypted representations are compositional only in the sense that “with the right highly nonlinear decoding scheme compositional representations can be extracted,” which is also true of some coding schemes typically interpreted as noncompositional, such as idiosyncratic representations of each input. That is, this case provides a clean example of a functionally-but-not-concatenatively compositional representation (cf. Van Gelder, 2014). This raises questions about if and when it is feasible to rigorously confirm whether a system's computations are compositional from representational analyses—or perhaps even about whether there can be a universal notion of compositionality below the computational level.

This example illustrates how opaque the relationship between the superficial format of representation and computation can be in the worst case. However, computing using homomorphic encryption is generally too slow for many practical uses, which perhaps hints at one of the practical constraints that pushes neural and machine learning systems toward simpler—and more aligned—representation formats; we will return to this question below.

## Discussion

In this work, we have discussed some of the issues that arise in trying to interpret a system via its representations. We focused primarily on issues that arise due to biases that emerge in the representations learned by deep neural models trained to perform simple tasks—biases that favor certain features over others. We also highlighted the possibility for strong dissociation between representations and computations, as in the case of homomorphic encryption. Here, we discuss some natural questions that arise from these results and how we see these issues relating to broader issues in the field.

### Should we interpret these representational effects as biases?

Above, we described the experimental results as representation “biases.” However, it is natural to ask whether this is an appropriate description. The patterns of bias are driven by real differences in the features being computed, and they have real downstream consequences (e.g., for the magnitude of representational perturbation needed to change the system's behavior). Thus, should we really interpret these effects as biases?

While we are sympathetic to this perspective, our central point is that these phenomena lead to biases in the inferences we draw about a system from using common representational analyses. Thus, whether these effects are interpreted as “biases” in the representations, they pose a challenge for interpreting frequently used analyses. We therefore see them as a useful case study in the broader issues in how we analyze representations to understand a system.

### Analyzing representations is still useful, even with biases—but complete understanding may be harder to achieve

It is important to note that these biases do not eliminate the possibility of achieving some understanding from representational analysis. For example, the easy linear features that are readily discoverable in the model’s representations play an important, causal role in the model's behavior. The fact that the multitask models and easy-task-only models appear very similar is precisely due to the fact that (some of) their computations are similar. Moreover, biases in representation can lead to downstream biases in subsequent learning (Lampinen et al., 2024; cf. Laamerad et al., 2024)—thus, representation biases can be important to how systems behave.

However, what the biases may limit is our ability to readily achieve complete understanding. If representations are biased away from certain features, then some important aspects of a system's behavior may be difficult to detect in its representations without a priori knowledge of what features the system is representing. For example, the analyses used to quantify the role of different ways of representing the same nonlinear feature relied on knowing all the possible patterns of representing the feature in question (or rather, an augmented representation that is linearly transformable to any possible pattern of representation), which is clearly impractical for natural systems in most cases.

These types of effects may result in a kind of “streetlight effect” where the field as a whole focuses on certain representational features that can be more reliably detected as strong signals in the neural activity and ignores others that may nevertheless be important in the system's computations and behavior. This problem may be exacerbated if the easiest-to-detect signals also happen to be the simplest to interpret.

### What are the potential solutions?

It is natural to ask whether alternative metrics for comparing systems could address some of these challenges. While considering a range of metrics is a generally good practice—especially since the optimal metrics may differ across experimental settings (Bobadilla-Suarez et al., 2020)—it is not clear that there is a complete solution from metrics alone. First, there is often convergence of conclusions across different types of metrics (Feather et al., 2025), as well as theoretical results showing alignment (Williams, 2024), even between seemingly dissimilar metrics (Harvey et al., 2024) in some cases. Indeed, Lampinen et al. (2024) found that the representation biases they observed produced similar biases in analysis results even when using less clearly variance-driven metrics, such as cosine similarity. Moreover, even metrics that impose stronger constraints on the mapping between systems, such as soft matching distance (Khosla and Williams, 2024), are vulnerable to similar issues—insofar as they still rely on variance-impacted measures like correlation at the unit level. Of course, it would be possible to ignore the variance carried by a signal when analyzing representations, but this would simply introduce another large bias—many components in the model's representations are simply noise (in fact, all but two dimensions must ultimately be behavior-irrelevant in the simple model depicted above, due to its limited output rank). Thus, it will not necessarily be simple to address these issues by altering metrics alone. Nevertheless, we believe that exploring whether modified metrics can help to ameliorate these issues is a valuable direction for future work.

We add one further comment specifically on the case of linear decoding analyses. In principle, unregularized linear decoding is invariant to signal magnitude in the input space; this is in contrast to its strong sensitivity to signal variance in the output space, as in encoding analyses. However, even for decoding analyses, variance biases reappear in the presence of regularization, either explicit regularization such as ridge or lasso regression or implicit regularization or biases in the optimization dynamics of nonlinear decoders (Neyshabur et al., 2014; Hermann et al., 2024). Furthermore, noise in the representations will disproportionately distort low-variance signals (which is, of course, a key motivation for regularization), and noise can therefore contribute to biases even from unregularized linear regression. Thus, while decoding analyses may in some cases avoid some of these biases, in most cases they are not completely immune.

Second, as always, causal interventions provide stronger confirmation of understanding. However, causal experiments do not fully address these issues on their own. Even in cases where causal interventions target unit- or population-level representations, these targets that we select for intervention are generally those that we have already identified using tools such as regression, PCA, or clustering—and thus biases in these tools will mean that even if we verify the causal role of the representational features we identify, we still may not have identified the complete set of representational features that play a causal role in behavior. This problem is exacerbated by the possibility of causal preemption (cf. Mueller, 2024): strong interventions on a particular representational feature may effectively overwhelm the causal contribution of other features, leading us to believe we have identified the only mechanism that mediates behavior, when in fact we have only identified one of multiple mechanisms that contribute. Verifying causality strengthens our conclusions about the contributions of a mechanism but does not (on its own) guarantee that our understanding is complete.

Thus, one key to resolving the issues raised here is a strong focus on completely characterizing behavior (cf. Krakauer et al., 2017) and the causal role that representations play in it. The feature biases we describe are defined precisely with respect to the behavioral outputs of the system. Thus, in the simple settings we consider, it would be easy to see that the strongly represented components do not causally mediate some of the system's behaviors, and subsequently it might be possible to reverse-engineer how the system was representing them. Of course, for natural systems (with much richer input and output spaces), it may not be so easy to characterize behavior fully—nor to verify the causal role of a pattern of neural activity. Nevertheless, focusing on characterizing all the representational features that contribute to behavior will help to address these issues—especially if they are characterized across a broad range of varied settings (incorporating systematic manipulations) that may alter how computational mechanisms are engaged (Cisek and Green, 2024; Carvalho and Lampinen, 2025).

### If representations need not align, why do we often observe convergence?

Seeing that representations may be biased—or even encrypted—in ways that dissociate from their computational role makes it potentially more interesting that in practice we often observe representational alignment between systems (Khaligh-Razavi and Kriegeskorte, 2014; Yamins et al., 2014; Schrimpf et al., 2021; Nayebi et al., 2023; Huh et al., 2024; Sucholutsky et al., 2025). One possible explanation is that the systems are primarily converging on the easiest features, which dominate the representations of all systems, regardless of whether those features are most important to the systems’ computations.

However, a more optimistic possibility is that this convergence reflects real constraints on the types of representations that learning systems will tend to arrive at, for example, constraints on the solutions that are efficiently computable, on the solutions that satisfy a behavioral constraint (Marchetti et al., 2024; Piantadosi and Gallistel, 2024), or on the solutions that learning dynamics yield (Huh et al., 2021; Smith et al., 2021; Saxe et al., 2022). In this case, it may be productive to study more deeply the types of shared constraints on representation learning that drive alignment across systems (Cao, 2022a; Cao and Yamins, 2024; cf. Huh et al., 2024)—and across scales of analysis (Guest and Love, 2017).

### The mutual benefits of knowledge sharing between neuroscience and machine learning

At a higher level, we believe that these results illustrate the broader point that interactions between machine learning and neuroscience can be mutually beneficial. The ability to completely control the training of machine learning models, and to causally intervene on every component of them, allows us to explore issues like the reliability and faithfulness of analyses used in neuroscience (cf. Jonas and Kording, 2017) and offers opportunities for exploring underlying assumptions. Reciprocally, neuroscience has a substantial history of analyzing complex systems—and identifying the challenges of bridging between levels of analysis—that is useful context for understanding the shared challenges in interpreting machine learning models (cf. He et al., 2024).

## Synthesis

Reviewing Editor: Nicholas J. Priebe, The University of Texas at Austin Decisions are customarily a result of the Reviewing Editor and the peer reviewers coming together and discussing their recommendations until a consensus is reached. When revisions are invited, a fact-based synthesis statement explaining their decision and outlining what is needed to prepare a revision will be listed below. The following reviewer(s) agreed to reveal their identity: NONE.

Two reviewers evaluated the resubmitted manuscript "Representation biases: variance is not always a good proxy for importance."  Both reviewers agreed that the manuscript describes an important issue for the analysis of neuroscience data. They both agreed that the manuscript could be published as is. The reviewer comments on the manuscript are given below if the authors wish to make any changes to their manuscript.

Review #1:

This paper synthesizes recent machine learning work by others, as well as reviewing the broader literature, on a critical problem in neuroscience, assessing representations.

The core point is important and often neglected: variance-based analyses like PCA, RSA, and regression systematically favor simple linear features over complex nonlinear ones, even when both are equally critical computationally. Well-controlled simulations where the ground truth is known demonstrate these biases.

I found the discussion of how these biases can arise from learning dynamics to be interesting and informative. I think the authors are highlighting a critical and foundational issue in neuroscience that I have been interested in myself. I don't suggest any revisions but mention some loose connections below that may or may not be useful to the authors.

The problem identified seems to go well beyond neuroscience. The feature selection or creation problem in pre-deep learning ML mostly focused on metrics (linear correlation, chi-square tests) that neglected nonlinear feature interactions. For feature selection, the problem is the intractability of evaluating all feature subsets, which is a different viewpoint (building vs. evaluating representations) on a related problem.

At my new job at Los Alamos, I see this issue in evaluation all the time. Using MSE to evaluate match between physics foundational models and ground truth simulations of physics equations (computationally expensive) often shows terrific correspondence even when all the interesting high-frequency features are missed. I have distant memories of the same issues comparing learning curves of humans and cognitive models -- MSE would be dominated by the decrease in error rate as opposed to the more important details of which problems were most easily mastered.

I wonder if there's a connection here to short-cut learning? That seems like a case where both model learning dynamics and evaluation metrics share the same bias.

I am definitely not asking for cites, but some older work I was involved with may interest the authors.

Bobadilla-Suarez et al. (https://link.springer.com/article/10.1007/s42113-019-00068-5) takes a complementary approach worth considering: using decoding as ground truth (i.e., classifier confusion matrix) to evaluate which similarity measures best capture what's informationally relevant. There is no constraint on the form of the classifier.

Guest &Love (https://elifesciences.org/articles/21397) took the question considered by the authors and reversed it - the fact that we can retrieve similarity spaces from brain measures like fMRI implies a lot about neural representations, especially given many schemes (factorial, hash coding) would be invisible to our methods.

Brad Love

Review #2:

This paper reviews a series of findings that show some serious limitations on the ability of common representational analyses to shed light on computational similarities and differences between different systems. What the authors dub "representational biases" - for example, towards simpler features - might result from differences in the training curriculum, differences in the difficulty of the task. They result in double dissociations between the representational similarity of two systems and their computational similarity, so that systems performing the same task might look quite different from each other, while systems performing different tasks might look almost the same.

These results foreground an underappreciated question: why (and when) should we expect representational similarity to be a reliable guide to computational similarity? And to the extent that we cannot - because representational analyses fail to provide a complete picture of computational structure - the paper goes on to point out some pitfalls for research in neuroscientific modeling as well as the growing field of mechanistic interpretabilty in ML (and including causal interventions in both).

If the paper is to make the stronger point that sometimes seems implied: that representational analyses are consistently (and not just potentially) misleading about computational or functional similarity between systems, it would be helpful to address a few further questions. For example, beyond the one reference to Fel et al, do we have reason to believe that these results generalize to larger networks performing more difficult tasks? Or to networks that have just enough parameters to do the task(s), but no extra capacity?

If we think of computation as the manipulation of representations, step by step, then it would be natural to try to track the transformation of representations across layers (in a feedforward network) and time steps in order to gain a more complete picture of the sequence of operations a system was performing on its representations, and thus a better picture of what computations were being performed. (The author seems receptive to something along these lines, as they acknowledge that encoding analyses will necessarily be more biased towards input features than decoding analyses, which ought to track outputs, and thus tasks, better).

The homomorphic encryption example is of independent interest, as it shows how, in principle, representational analyses can be very weak indeed. For example, the author points out the difficulty of determining whether a system is compositional in such a case. (I think the point is rather stronger: it shows that compositionality itself may be nothing more than a matter of perspective. But this may be an idiosyncratic reaction on my part).
